# Apigenin alleviates TGF-β1-induced nasal mucosa remodeling by inhibiting MAPK / NF-kB signaling pathways in chronic rhinosinusitis

**DOI:** 10.1371/journal.pone.0201595

**Published:** 2018-08-30

**Authors:** Hyun-Woo Yang, Hwee-Jin Kim, Joo-Hoo Park, Jae-Min Shin, Heung-Man Lee

**Affiliations:** 1 Department of Biomedical Science, Korea University, College of Medicine, Seoul, Korea; 2 IVD Support Center, Korea University Guro Hospital, Korea University, College of Medicine, Seoul, Korea; 3 Department of Otorhinolaryngology-Head and Neck Surgery, Korea University, College of Medicine, Seoul, Korea; University of Insubria, ITALY

## Abstract

**Background:**

Chronic rhinosinusitis is involved in tissue remodeling of nasal mucosa such as nasal myofibroblast differentiation and extracellular matrix production. Apigenin (4’,5,7-trihydroxyflavone) is a bioflavonoid compound and has anti-tissue remodeling characteristics. The aims of this study were to evaluate the effect of apigenin on TGF-β1-induced myofibroblast differentiation and extracellular matrix accumulation and to determine the underlying mechanism.

**Methods:**

Nasal fibroblasts and *ex vivo* nasal inferior turbinate tissues were stimulated with TGF-β1 with or without apigenin. The expression levels of α-SMA, fibronectin and collagen type I were determined by real-time PCR, western blot and immunocytochemical staining. Mitogen-activated protein kinase (MAPK) phosphorylation induced by TGF-β1 were determined by western blot analysis. The transcriptional activity of NF-κB was measured by luciferase assay. Migration effects of fibroblasts were evaluated by wound scratch and transwell migration assay. Contractile activity was determined by collagen gel contraction assay.

**Results:**

The expression levels of α-SMA, fibronectin, and collagen type I significantly increased in TGF-β1-stimulated nasal fibroblasts. In TGF-β1-stimulated nasal fibroblasts, apigenin inhibited the expressions of α-SMA, fibronectin, and collagen type I. Inhibitors of MAPK (p-38, JNK) and NF-κB blocked the expression of α-SMA, fibronectin and collagen type I. Apigenin suppressed the activation of MAPK (p-38, JNK) and NF-κB induced by TGF-β1 treatment. Apigenin also inhibited the functional activity of fibroblasts by reducing the migration and collagen contractile activities.

**Conclusions:**

These results suggests the possible use of apigenin as a chronic rhinosinusitis therapeutic agent which can suppress tissue remodeling in nasal mucosa.

## Introduction

Chronic rhinosinusitis (CRS) is a multifactorial inflammatory disease in nose and paranasal sinuses and lasts more than 12 weeks. Nearly 10% of the world’s population has this disease, and those infections are accompanied by symptoms such as nasal obstruction, congestion, discharge, and facial pain [1, 2]. Despite this disease being widespread, the pathogenesis of CRS is not fully understood.

Tissue remodeling is known to be an important cause of CRS, characterized by overgrowth of nasal mucosa, basement membrane thickness, glandular hyperplasia, inflammatory cell infiltration, and mucosal fibrosis [2–5]. Tissue remodeling of CRS involves the expression of transforming growth factor β1 (TGF-β1), a pro-fibrotic cytokine that contributes to myofibroblast differentiation of nasal fibroblasts [6]. The myofibroblast is an activated fibroblast that expresses alpha-smooth muscle actin (α-SMA) and extracellular matrix (ECM) such as fibronectin and collagen type I, II, III, V, and XI [7]. Therefore, the fibroblast is an important target cell for the treatment of CRS, and the mechanism to prevent the differentiation of nasal fibroblast, can regulate CRS by suppressing ECM accumulation.

Apigenin (4 ', 5,7-trihydroxyflavone) is a flavonoid abundant in fruits, vegetables, herbs, and beverages [8]. In a recent study, it was reported that biological control could function as an anti-cancer and anti-inflammation [9–11]. Apigenin ameliorated fibrosis and ECM accumulation by bleomycin [12] and suppressed TGFβ1-induced myofibroblast differentiation in lung fibroblasts [13]. Apigenin can modulate collagenase, matrix metalloproteinase expression in dermal fibroblasts [14, 15] or regulate cell migration and invasion [16].

However, the anti-tissue remodeling effect of apigenin on nasal mucosa has not yet been demonstrated. Therefore, we examine whether apigenin inhibits nasal fibroblast activation and ECM remodeling in TGF-β1-induced fibroblasts and investigate its underlying mechanisms.

## Materials & methods

### Nasal fibroblast culture

Nasal fibroblasts were obtained from the inferior turbinate of patients with rhinoplasty (4 males and 2 females; mean age 34.7 ± 2.1). All patients were recruited from the Department of Otorhinolaryngology, Korea University Medical Center, Korea. We have received consent from all patients and have received a written consent. All patients were composed of adults over 20 years of age. They were recruited and approved by the Korea University Medical Center Institutional Review Board, which also authorized the research, which carried out in accordance with the guidelines of the Human Ethics Committee of the Korea University Guro Hospital. Patients were nonsmokers and had not been treated with oral or topical corticosteroids or antibiotics for at least 4 weeks before surgery. There were no known allergies, asthma, or aspirin sensitivity among the patients. Informed written consent was provided according to the Declaration of Helsinki. Inferior turbinate tissues were isolated by enzymatic digestion with collagenase (500 U/mL; Sigma-Aldrich, St Louis, MO), hyaluronidase (30 U/mL, Sigma-Aldrich), and DNase (10 U/mL, Sigma-Aldrich). Nasal fibroblasts were cultured in Dulbecco’s Modified Eagle Medium containing 10% heat-inactivated fetal bovine serum (Invitrogen, Carlsbad, CA), 10,000 μg/mL streptomycin (Invitrogen), and 1% 10,000 U/mL penicillin (Sigma-Aldrich). The purity of the obtained nasal fibroblasts was confirmed by characteristic spindle-shaped cell morphology and flow cytometry [17].

### Cytotoxicity assay

In order to determine the cytotoxicity of apigenin, IncuCyte^®^ Cytotoxicity Assay (Essen BioScience Inc., Ann Arbor, MI) was performed. Nasal fibroblasts of 4 x 10^5^cells/ml were seeded into 96-well tissue culture plates. After seeding, the medium was replaced with apigenin and treated with IncuCyte^®^ Cytotox Green Reagent (Essen BioScience Inc.). The viability of the fibroblasts was measured for 72 hours at 37°C in an atmosphere of 5% CO_2_ and 95% humidity. Apoptotic fibroblasts were stained by IncuCyte^®^ Cytotox Green Reagent. The viability of fibroblasts was calculated by measuring green fluorescence through IncuCyte^®^ software (Essen BioScience Inc.).

### Real-time PCR

To evaluate levels of *a-SMA*, *fibronectin*, and *collagen type I* expression, real-time PCR was performed. Total RNA was extracted by a Trizol reagent (Invitrogen). The synthesis of cDNA proceeded with Maxime RT PreMix kit (Intron Biotechnology, Korea) according to the manufacturer’s protocol. PCR was performed using the following primers: *a-SMA* (sense sequence 5’-ACT CAC CTC TTC AGA ACG AAT TG-3’ antisense sequence 5’-CCA TCT TTG GAA GGT TCA GGT TG-3’), *fibronectin* (sense sequence 5’-GGA TGC TCC TGC TGT CAC-3’, antisense sequence 5’-CTG TTT GAT CTG GAC CTG CAG-3’) *collagen type I* (sense sequence 5’-GGA TGC TCC TGC TGT CAC-3’, antisense sequence 5’-CTG TTT GAT CTG GAC CTG CAG-3’), and *GAPDH* (sense sequence 5’-GTG GAT ATT GTT GCC ATC AAT GAC C-3’, antisense sequence 5’-GCC CCA GCC TTC TTC ATG GTG GT-3’). Amplification reactions were performed as follows: an initial 2 minute denaturation step at 94°C; 40 cycles of 94°C for 5 seconds, 60°C for 10 seconds, 72°C for 20 seconds. All reactions were performed in a 20 μL volume. Real-time PCR was carried out in a QuantStudio 3 (Applied Biosystems, Foster City, CA) using 100 ng of cDNA template, 400 nM of each primer, and 10 mL of Power SYBR Green PCR Master Mix (Applied Biosystems) in a total volume of 20 μL. Analysis of relative gene expression was conducted using the 2(2DDCt) method. Each experiment was repeated at least 3 times, and *GAPDH* was used as an internal control.

### Western blot analysis

Nasal fibroblasts were seeded into a 60 mm culture dish with 5×10^5^ cells/mL and lysed in Ripa buffer (Sigma-Aldrich) with protease inhibitors (Sigma-Aldrich) and phosphatase inhibitors (Sigma-Aldrich). Proteins were separated via 10% SDS-polyacrylamide gel electrophoresis and transferred onto polyvinyl difluoride membranes (Millipore Inc., Billerica, MA). Membranes were blocked with a 5% bovine serum albumin. The blots were incubated with primary antibodies against α-SMA (Millipore Inc.), fibronectin (Santa Cruz Biotecknology Inc., CA), collagen type I (Abcam, Cambridge, UK), phospho-p38, total-p38, phospho-JNK, total-JNK, phospho-ERK, total-ERK (Cell Signaling, MA), total-p50, phospho-p50 (Santa Cruz Biotecknology Inc.), and β-actin (Santa Cruz Biotecknology Inc.) overnight at 4°C. HRP-conjugated secondary antibodies was incubated for 1 hour at room temperature and detected with Amersham imager 600 (GE healthcare, little Chalfont, UK)

### Immunocytochemical staining

To evaluate the protein expression and localization, Immunocytochemical staining was performed. Nasal fibroblasts were seeded on a cell culture slide (SPL Life Sciences, Korea) with 5×10^4^ cells/mL. Before using the cell culture slide, poly-L-lysine was used to coat the culture slide for 4 hours, after which it was exposed to UV light for 6 hours. Fibroblasts were pretreated with or without MAPK inhibitors (SB203580, SP600125, U0126) and NF-kB inhibitor (BAY11-7082) and stimulated with apigenin (5 μM) for 72 hours. Fibroblasts were fixed with 4% paraformaldehyde and permeabilized with 0.01% Triton X-100 in 1% bovine serum albumin for 10 minutes. After that, fibroblasts were blocked with 3% bovine serum albumin for 1 hour, incubated with primary antibodies against phospho-p50, and then incubated with anti-rabbit Alexa 555 secondary antibodies (Invitrogen). Finally, fibroblasts were counterstained with 4'-6-diamidino-2-phenylindole for 10 minutes. Stained fibroblasts were visualized using a confocal laser scanning microscope (LSM700, Zeiss, Oberkochen, Germany).

### Luciferase assay

To assess the promoter activity of NF-kB, luciferase assay was performed. Reporter gene of NF-kB (luc2P/NF-kB-RE/Hygro and hRluc) (Promega, Madison, WI) was transfected into nasal fibroblasts using Lipofectamine 2000 (Qiagen, Valencia, CA) in serum free DMEM media for 6 hours. After that serum free media was changed with DMEM media containing 10% fetal bovine serum and incubated for 24 hours. Transfected nasal fibroblasts were pretreated with MAPK or NF-kB inhibitors and stimulated with TGF-β1 for 1 hour. Relative activity of NF-kB promoter was calculated relative to Renilla activity using luminometer (Promega).

### Collagen measurements

To evaluate secreted soluble collagen, sircol collagen assay (Biocolor, Belfast, UK) was performed. Nasal fibroblasts were incubated with TGF-β1 with or without specific inhibitor or apigenin. The 500ul of supernatants in nasal fibroblast culture were mixed with 500ul of dye reagent containing Sirius red for 20 minutes. After that, samples were centrifuged for 20 minutes at 13,000 rpm. Collagen-dye complex was released by 200ul alkali reagent containing 0.5M sodium hydroxide. Concentration of soluble collagen was determined by spectrophotometry at 550 nm (Beckman Coulter, Fullerton, CA).

### Wound scratch assay

A wound scratch assay was performed to evaluate the migration effect of apigenin in nasal fibroblasts. Nasal fibroblasts were seeded into 6-well culture plate with 3×10^5^ cells/mL and grown until 90% confluence as a monolayer in DMEM media supplemented with 10% fetal bovine serum, 1% 10,000 U/mL penicillin, and 10,000 μg/mL streptomycin (Invitrogen). Nasal fibroblasts were starved with serum-free medium for 24 hours. After this period, the medium was replaced, and mitomycin C (5 μg/mL) was added to suppress the proliferation of nasal fibroblast for 15 minutes. A wound scratch was made using a 200 μl pipette tip. Nasal fibroblasts were pretreated with apigenin (10 μM) for 1 hour and then stimulated with TGF-β1 (5 ng/ml) for up to 48 hours. The results were viewed by fluorescence and bright field microscopy (Olympus BX51; Olympus, Tokyo, Japan). Wound closure was calculated using ImageJ software (National Institutes of Health). The percentage of wound closure is presented graphically.

### Transwell invasion assay

A Transwell invasion assay was performed to observe the invasion of nasal fibroblasts. Fibroblasts were seeded into a 6.5 mm insert (with 8.0 μm pore) on a 24-well plate with 5×10^4^ cells/mL and grown to 80% confluence in complete DMEM. After that, cells were starved with serum-free medium for 24 hours, and the medium at the bottom of the plate was replaced with a medium which either contained the apigenin (10 μM) or not for 1 hour. After this process, TGF-β1 (5 ng/ml) was applied for stimulation of nasal fibroblasts for up to 72 hours, and the medium was aspirated from the apical inserts and receiver well. To eliminate the medium, cells were washed in phosphate buffered saline 2–3 times. Invasion of nasal fibroblasts was confirmed with ‘Diff-Quick’ staining. Fixation was performed using “Diff-Quick” Fixative for 10 minutes, and cell staining and counterstaining were performed using “Diff-Quick” solutions I and II for 10 minutes, respectively. The results were analyzed by fluorescence and bright field microscopy (Olympus BX51; Olympus, Tokyo, Japan), and the number of transferred nasal fibroblasts was calculated by ImageJ software (National Institutes of Health, Bethesda, Maryland)

### Collagen gel contraction assay

To evaluate the contractile activity of nasal fibroblast, collagen gel contraction assay was performed. Type I collagen (Rat-tail; Bedford, MA) was diluted with 0.1% acetic acid to make a concentration of 3 mg/ml. 400ul of Serum free DMEM and 200ul of 3 mg/ml type I collagen were mixed and added 1M NaOH. Detached nasal fibroblasts was suspended in serum free media to 3.5 x 10^5^ cells/ml of density and mixed with neutralized collagen. The fibroblast-collagen mix liquid was seeded to 24-well plate and incubated for 20 minutes. After that, polymerized gels were detected and added DMEM media containing specific inhibitor or apigenin and stimulated with TGF-β1 for 72 hours. Gel surface area was calculated with an ImageJ analyzer (National Institutes of Health).

### *Ex vivo* organ culture

Nasal inferior turbinate tissues were cut into 2 - 3mm^3^ pieces under sterile conditions. Before proceeding with the culture, the tissues were washed 3 times with phosphate buffered solution. Nasal inferior turbinate tissues were cultured in complete DMEM supplemented with 2% fetal bovine serum (invitrogen). The tissue fragments were placed on the upper transwell. The mucosal side was placed facing up, and the submucosal side was placed facing down. Each tissue was placed into 6-well plates, and DMEM was added. Each tissue was pretreated with or without MAPK inhibitors (SB203580, SP600125) and NF-kB inhibitor (BAY11-7082) and then stimulated with apigenin (5 μM) for 72 hours. Nasal inferior turbinate tissues were used to detect the mRNA and protein levels of *a*-SMA, fibronectin and collagen type I.

### Statistical analysis

The statistical significance of the difference between the control and experimental data was analyzed using Wilcoxon’s signed-rank test. A p-value < 0.05 was accepted as statistically significant. The results were obtained from at least 3 independent experiments. The statistical significance of the differences between control and experimental data was analyzed using the unpaired t-test or one-way analysis of variance (ANOVA) followed by Tukey’s test (GraphPad Prism, version 5, GraphPad Software, San Diego, CA). Significance was established at the 95% confidence level. P-values <0.05 were accepted as statistically significant.

## Results

### Cytotoxic effect of apigenin in nasal fibroblasts

**Fig 1:**
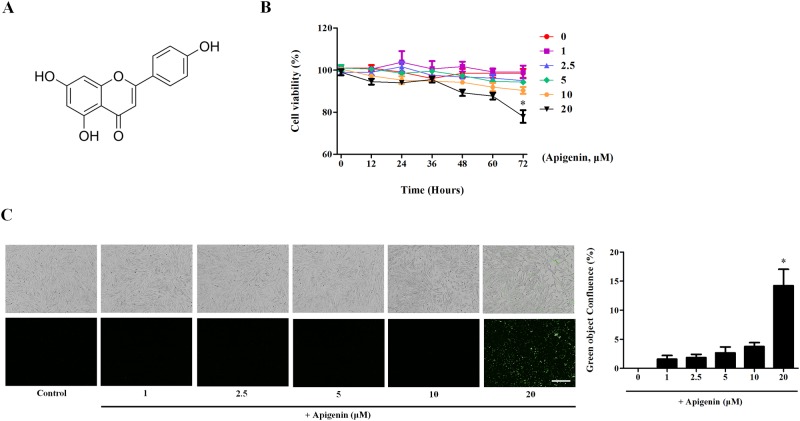
Cytotoxicity effect of apigenin in nasal fibroblasts. The cytotoxicity test was performed using the IncuCyte^®^ real-time detection system. (A) Structure of apigenin. (B) Nasal fibroblasts were treated with apigenin for 72 hours. Fibroblast viability was calculated by IncuCyte^®^ software. (C) Phase contrast-fluorescence images were taken at 72 hours by IncuCyte^®^. The graph was calculated by measuring the green object confluence. Scale bar = 300 μm. Results were obtained from at least three independent experiments.

Apigenin (4′,5,7-trihydroxyflavone) is a plant flavonoid (Fig 1A). The real-time IncuCyte^®^ assay was performed to confirm the cytotoxicity of apigenin. Apigenin was applied to nasal fibroblasts at a concentration of 0–20 μM for 72 hours. Apigenin did not show cytotoxicity up to a concentration of 10 μM. However, fibroblast death was induced when treated with 20 μM for 72 hours (Fig 1B). Apigenin and IncuCyte^®^ Cytotox Green Reagent (250 nM) were applied together to confirm the death rate of the fibroblasts, which was confirmed by IncuCyte^®^ (Fig 1C). Green object confluence increased by 14.2% in the 20 μM apigenin treatment compared to the control. Therefore, apigenin have not cytotoxic at less than 20 μM.

### Apigenin alleviates TGF-β1-induced myofibroblast differentiation in nasal fibroblasts

**Fig 2:**
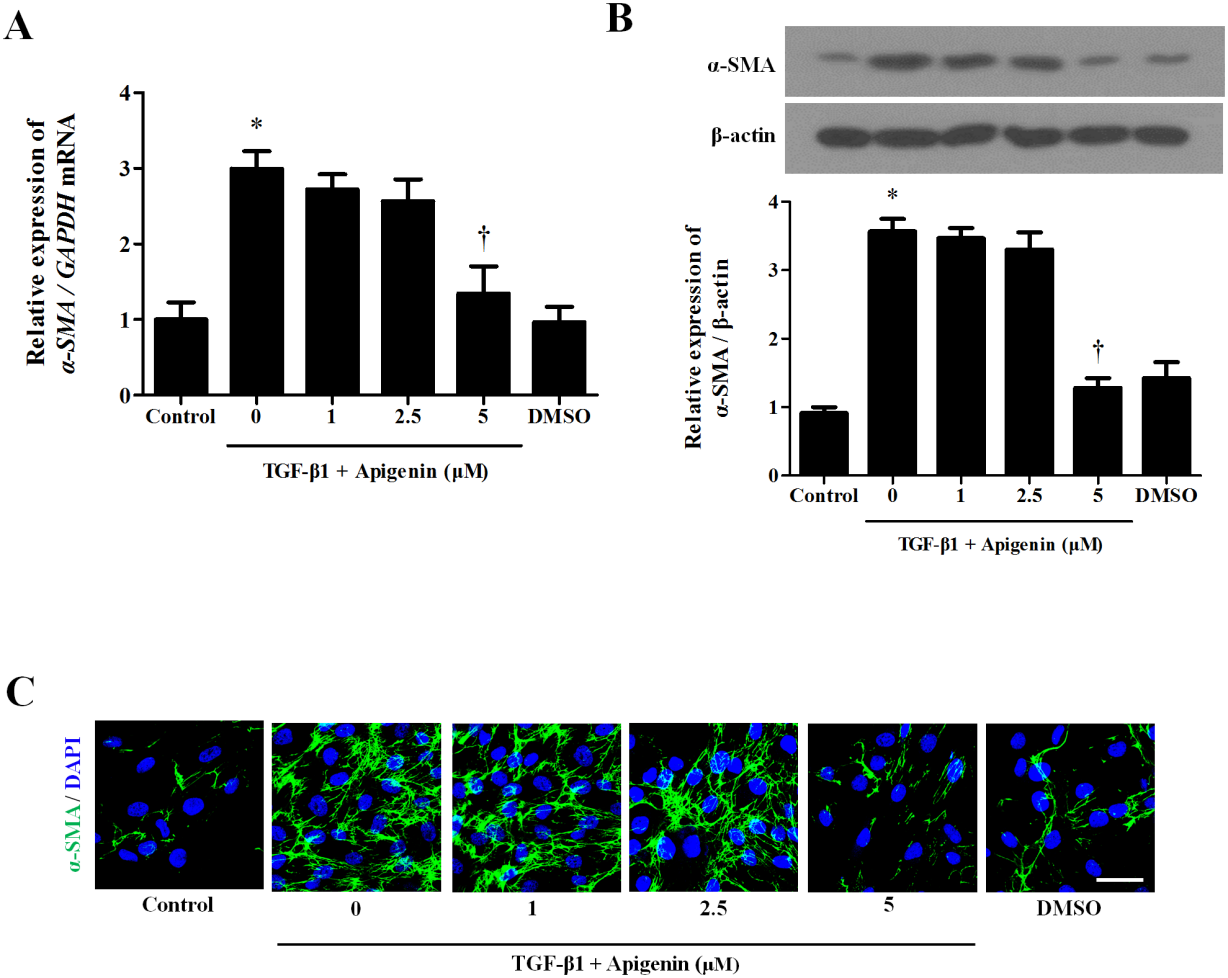
Effect of apigenin on myofibroblast differentiation induced by TGF-β1 in nasal fibroblasts. Nasal fibroblasts were pretreated with apigenin for 1 hour and combined with TGF-β1. (A) Messenger RNA expression level of *a-SMA* was determined by real-time PCR. (B) Protein expression level of *a*-SMA was evaluated by western blot. (C) Immunofluorescence images were obtained by a confocal microscope. Immunocytochemical images are shown with *a*-SMA (green) and DAPI (blue). Scale bar = 100 μm. Results were obtained from at least three independent experiments. * *p* <0.05 vs. TGF-β1; ^†^*p* < 0.05 vs. apigenin.

TGF-β1 treatment induces myofibroblast differentiation of nasal fibroblasts. To examine whether apigenin has an inhibitory effect on myofibroblast differentiation induced by TGF-β1, apigenin was pretreated for 1 hour and then stimulated with TGF-β1. TGF-β1 significantly increased mRNA and protein expression of *a*-SMA, a myofibroblast marker. Apigenin did not inhibit the expression of *a*-SMA by TGF-β1 at a concentration up to 2.5 μM, whereas 5 μM of apigenin significantly inhibited *a*-SMA expression (Fig 2A and 2B). In immunocytochemical staining, the expression of *a*-SMA in the cytoplasm induced by TGF-β1 was significantly decreased by 5 μM of apigenin (Fig 2C).

### Apigenin inhibits TGF-β1-induced extracellular matrix production in nasal fibroblasts

**Fig 3:**
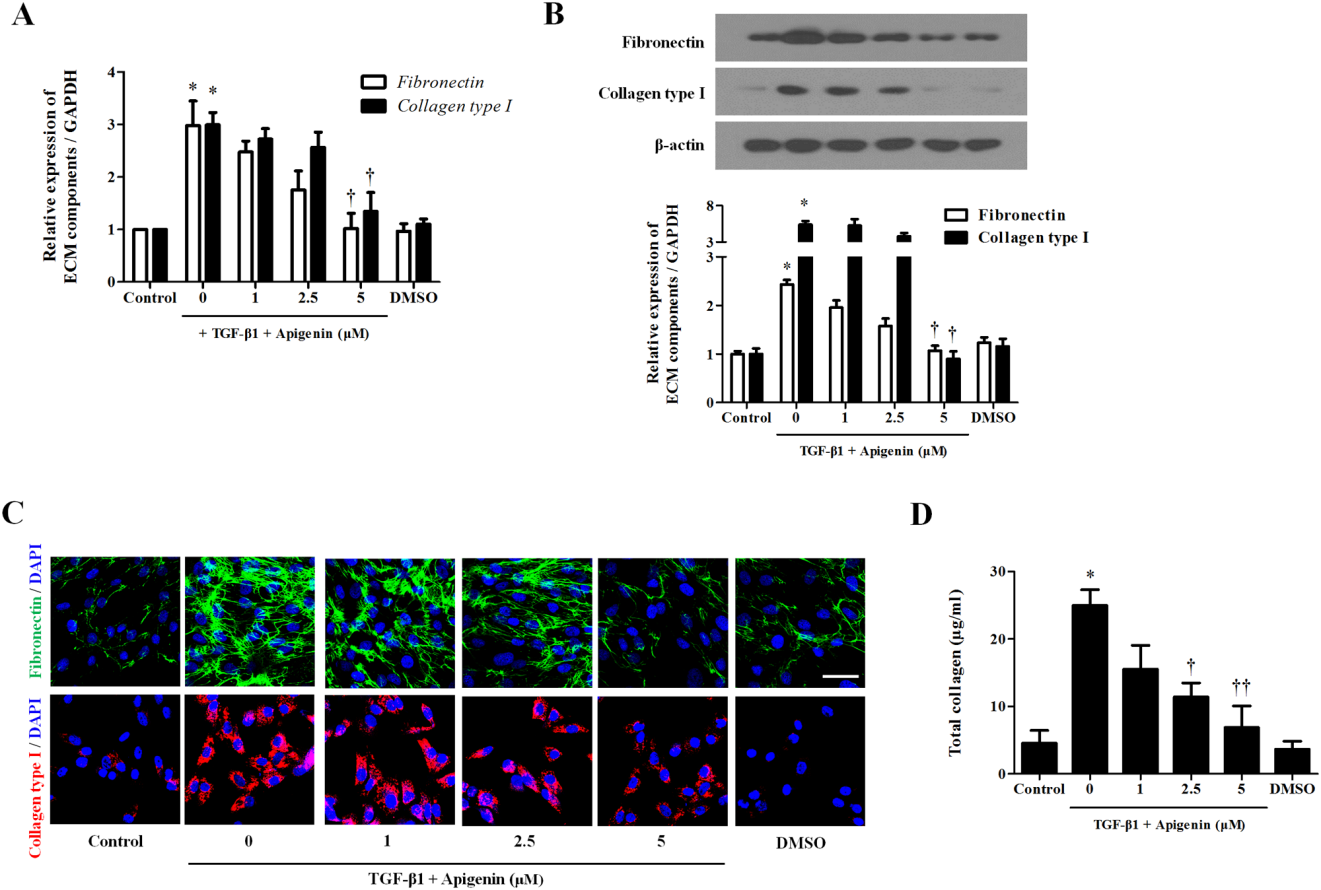
Effect of apigenin on extracellular matrix production induced by TGF-β1 in nasal fibroblasts. Nasal fibroblasts were pretreated with apigenin for 1 hour and combined with TGF-β1. (A) Messenger RNA expression levels of *fibronectin* and *collagen type I* were determined at 48 hours by real-time PCR. (B) Protein expression levels of fibronectin and collagen type I were evaluated by western blot. (C) Immunofluorescence images were obtained by a confocal microscope. Immunocytochemical images are shown with fibronectin (green), collagen type I (red), and DAPI (blue). Scale bar = 100 μm. Results were obtained from at least three independent experiments. (D) Total collagen was evaluated by sircol assay. * *p* <0.05 vs. TGF-β1; ^†^*p* < 0.05, ^††^*p* < 0.01 vs. apigenin.

To determine whether apigenin has an inhibitory effect on TGF-β1-induced ECM in nasal fibroblasts, apigenin was pretreated for 1 hour and then stimulated with TGF-β1. TGF-β1 significantly increased mRNA and protein expression of ECM components such as fibronectin and collagen. Apigenin did not inhibit the expression of fibronectin and collagen by TGF-β1 at a concentration up to 2.5μM, but 5μM of apigenin significantly inhibited fibronectin and collagen expression (Fig 3A and 3B). In immunochemical staining, expression of fibronectin and collagen induced by TGF-β1 was significantly decreased in 5 μM of apigenin (Fig 3C). Total soluble collagen was measured by sircol assay. TGF-β1 significantly increased total collagen, and apigenin had an inhibitory effect at concentrations above 2.5 μM (Fig 3D).

### Apigenin suppresses TGF-β1-induced phosphorylation of MAPK in nasal fibroblasts

**Fig 4:**
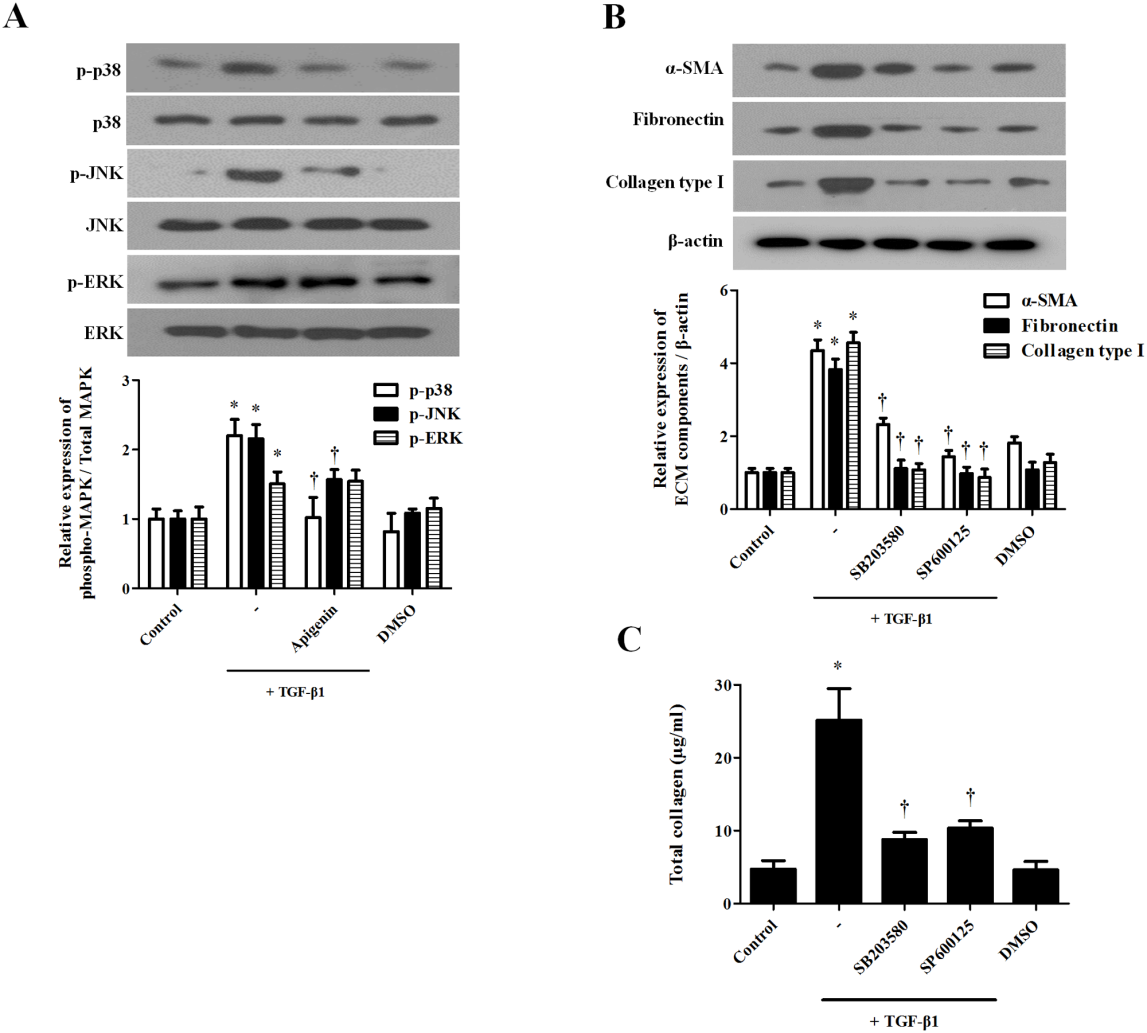
Effect of apigenin on the TGF-β1-induced MAPK signaling pathway in nasal fibroblasts. Nasal fibroblasts were pretreated with apigenin (5 μM) for 1 hour and combined with TGF-β1 (5 ng/ml). (A) Phosphorylation of MAPK (p-p38, p-JNK, and p-ERK) was detected by western blot. (B) Nasal fibroblasts were stimulated with TGF-β1 with or without the following specific inhibitors: SB203580 (10 μM), SP600125 (5 μM). Protein expression levels of *a*-SMA, fibronectin, and collagen type I were determined by western blot. (C) Total collagen was evaluated by sircol assay. Results were obtained from at least three independent experiments. * *p* <0.05 vs. TGF-β1; ^†^*p* < 0.05 vs. apigenin.

TGF-β1 activates the MAPK signaling pathway and is associated with myofibroblast differentiation and ECM production. Apigenin (5 μM) was pretreated for 1 hour and stimulated with TGF-β1 for 30 minutes. Western blotting was performed to confirm that apigenin inhibited TGF-β1-activated phosphor-p38, JNK, and ERK. Apigenin significantly inhibited TGF-β1-induced phosphorylation of p38 and JNK, but not ERK phosphorylation (Fig 4A). To determine whether phosphorylation of p38 and JNK affects myofibroblast differentiation and ECM production of nasal fibroblasts, each specific inhibitor was pretreated for 1 hour and applied with TGF-β1 for 72 hours. The protein expressions of *a*-SMA, fibronectin, and collagen were determined by western blot. P38 inhibitor (SB203580) and JNK inhibitor (SP600125) significantly inhibited TGF-β1-induced *a*-SMA, fibronectin, and collagen (Fig 4B). Total soluble collagen induced by TGF-β1 was significantly decreased by p38 and JNK inhibitor (Fig 4C).

### Apigenin inhibits TGF-β1-induced NF-kB signaling pathway in nasal fibroblasts

**Fig 5:**
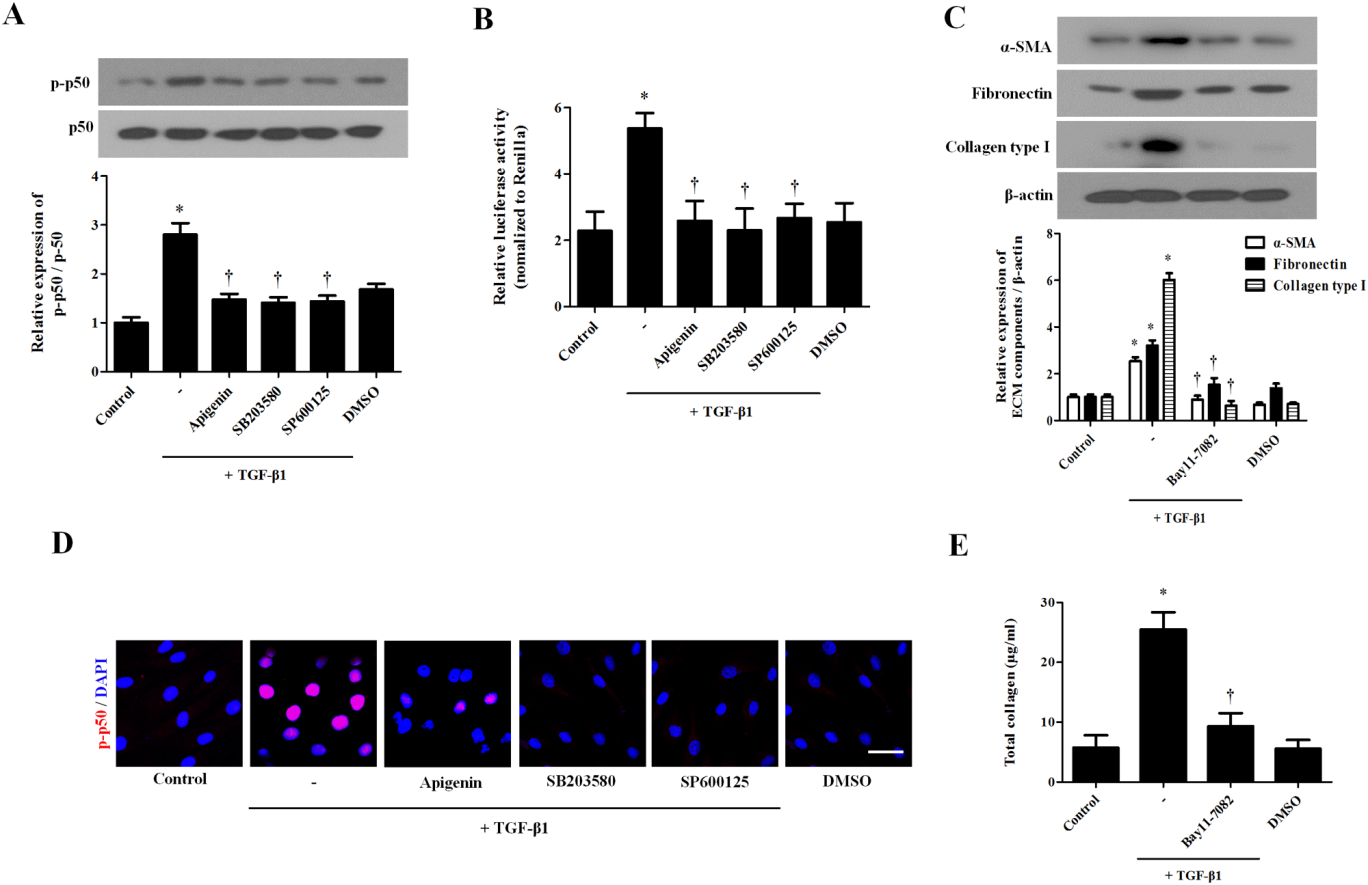
Effect of apigenin on TGF-β1-induced NF-κB activation in nasal fibroblasts. Nasal fibroblasts were pretreated with the following drugs and specific inhibitors: apigenin (5 μM), SB203580 (10 μM), and SP600125 (5 μM) and then stimulated with TGF-β1. (A) Phosphorylation of p50 was determined by western blot. (B) The p50 promoter activity was assessed by luciferase assay. Nasal fibroblasts were pretreated with NF-κB (BAY11-7082, 3 μM) and with TGF-β1 for 72 hours. (C) Protein expression levels of *a*-SMA, fibronectin, and collagen type I were determined by western blot. (D) Immunofluorescence images were obtained by a confocal microscope. Immunocytochemical images are shown with p50 (red) and DAPI (blue). Scale bar = 100 μm. (E) Total collagen was evaluated by sircol assay. The results were obtained from at least three independent experiments. * *p* <0.05 vs. TGF-β1; ^†^*p* < 0.05 vs. apigenin.

TGF-β1 induces activation of NF-kB and induces myofibroblast differentiation and ECM component production. TGF-β1 induced phosphorylation of p50, whereas apigenin, and P38, and JNK inhibitor inhibited phosphorylation of p50 by TGF-β1 (Fig 5A). The promoter activity of p50 was confirmed by luciferase assay. TGF-β1 significantly increased p50 promoter activity, while apigenin and p38 and JNK inhibitors inhibited promoter of p50 activation by TGF-β1 (Fig 5B). To investigate the effect of the NF-kB pathway on the production of myofibroblast differentiation and ECM components, NF-kB inhibitor (BAY11-7082) was pretreated with NF-kB inhibitor for 1 hour and stimulated with TGF-β1 for 72 hours. The protein expressions of *a*-SMA, fibronectin, and collagen type 1were determined by western blot. NF-κB inhibitor significantly inhibited TGF-β1-induced *α*-SMA, fibronectin, and collagen type I expression (Fig 5C). In immunochemical staining, TGF-β1 induced the expression of p-p50 protein and translocation into the nucleus. Apigenin and p38 and JNK inhibitors inhibited the expression and translocation of p-p50 (Fig 5D). Total collagen increased after treatment with TGF-β1 and was reduced by apigenin and P38 and JNK inhibitors (Fig 5E).

### Apigenin inhibits TGF-β1-induced migration and collagen gel contraction in nasal fibroblasts

**Fig 6:**
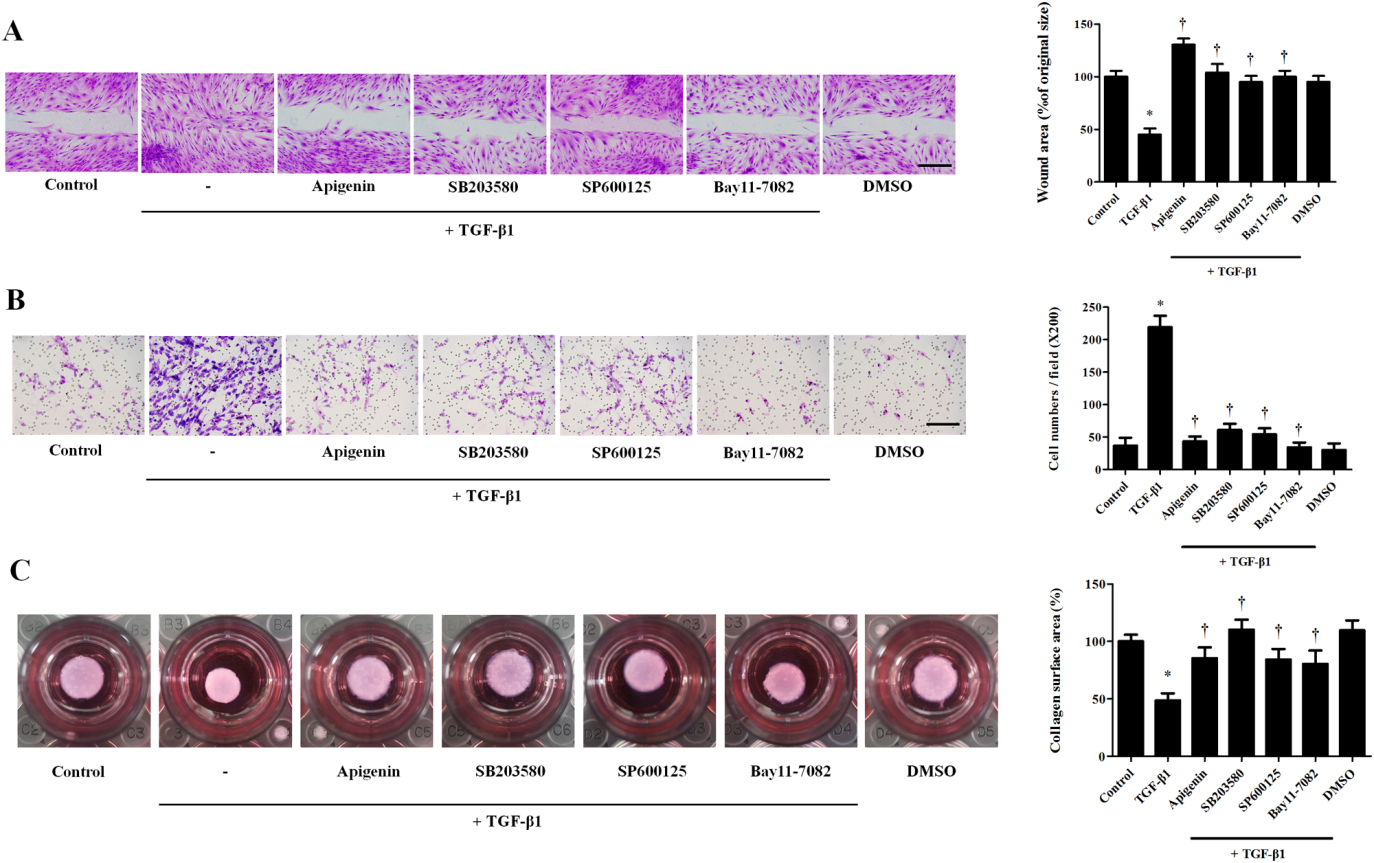
Effect of apigenin on TGF-β1-induced migration and gel contraction in nasal fibroblasts. Nasal fibroblasts were pretreated with the following drugs and specific inhibitors: apigenin (5 μM), SB203580 (10 μM), SP600125 (5 μM), and BAY11-7082 (3 μM) and then stimulated with TGF-β1 (5 ng/ml). (A) Migration of fibroblasts was assessed by a wound scratch assay. The images were obtained by a bright field microscope. The wound area was measured using an ImageJ analyzer. (B) Invasion of fibroblasts was assessed by a transwell invasion assay. The number of fibroblasts was measured using an ImageJ analyzer. (C) Contractile activity was assessed by collagen gel contraction assay. The contraction area was measured using an ImageJ analyzer. Results were obtained from at least three independent experiments. * *p* <0.05 vs. TGF-β1; ^†^*p* < 0.05 vs. apigenin.

To investigate the effect of apigenin on migration and collagen gel contraction, wound scratch assay, Transwell^®^ invasion assay, and collagen gel contraction assay were performed. TGF-β1 treatment significantly increased fibroblast migration compared to the control group. SB203580, SP600125, BAY11-7082, and apigenin inhibited TGF-β1-induced migration (Fig 6A). In the transwell invasion assay, the number of migrated nasal fibroblasts significantly increased in the TGF-β1-treated group compared to the control group. Pretreatment with SB203580, SP600125, BAY11-7082, and apigenin prevented the TGF-β1-induced migration (Fig 6B). TGF-β1 treatment significantly decreased the surface area of collagen gels, and pretreatment with SB203580, SP600125, BAY11-7082, and apigenin prevented periostin-induced collagen contraction (Fig 6C).

### Apigenin inhibit TGF-β1-induced myofibroblast differentiation and ECM production in *ex vivo* organ culture of the inferior turbinate

**Fig 7:**
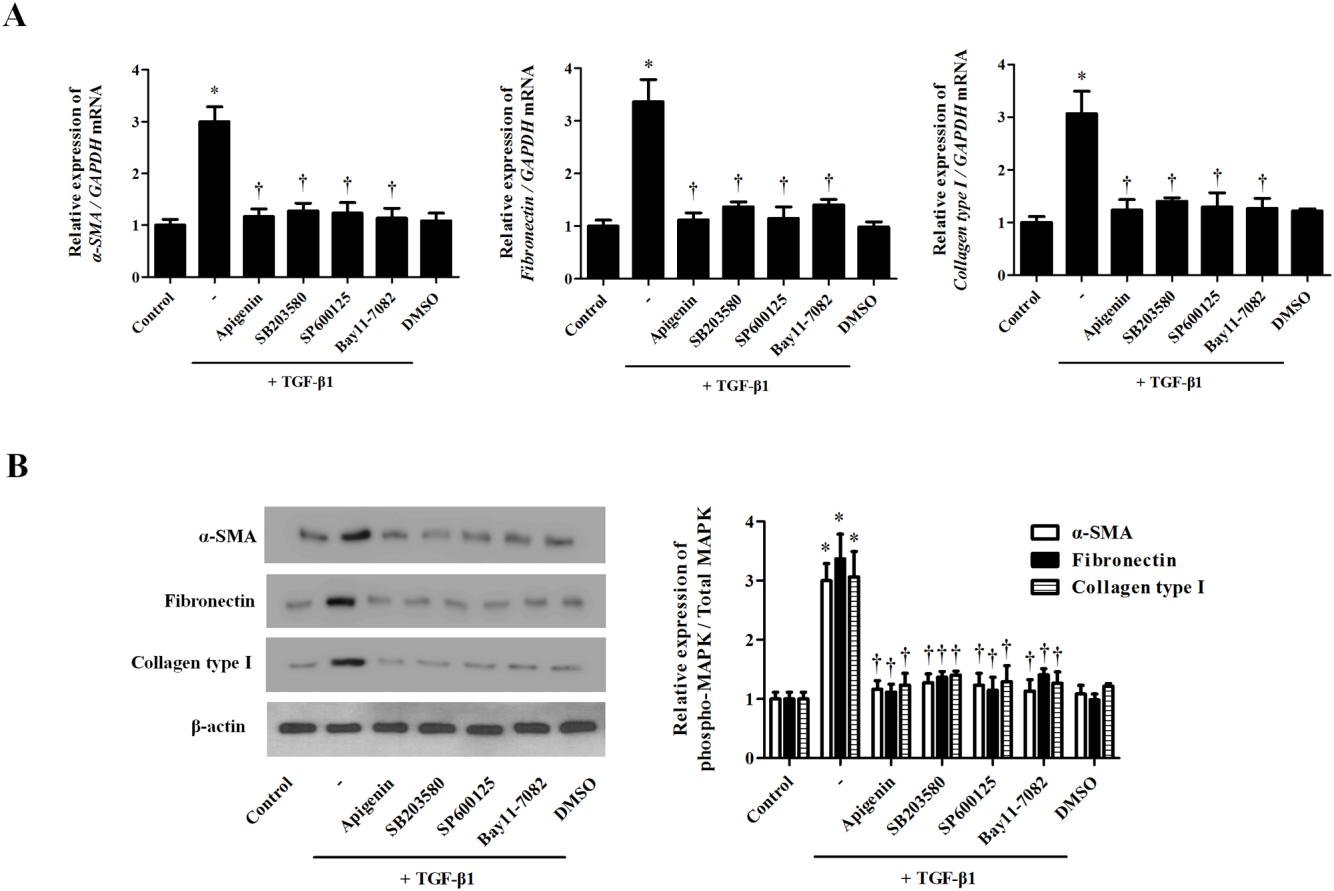
Effect of apigenin on TGF-β1-induced myofibroblast differentiation and extracellular matrix production in *ex vivo* nasal inferior turbinate tissues. Nasal fibroblasts were pretreated with the following drug and specific inhibitors: apigenin (10 μM), SB203580 (20 μM), SP600125 (10 μM), and BAY11-7082 (6 μM) and then stimulated with TGF-β1 (5 ng/ml). (A) The mRNA levels of *a-SMA*, *fibronectin*, and *collagen type I* were determined by real-time PCR. (B) The protein expressions of *a*-SMA, fibronectin, and collagen type 1 were assessed by western blot. Results were obtained from at least three independent experiments. * *p* <0.05 vs. TGF-β1; ^†^*p* < 0.05 vs. apigenin.

To confirm the effects of apigenin in nasal tissue, we cultured an *ex vivo* nasal inferior turbinate organ using an air-liquid interface culture method. TGF-β1 significantly increased *a*-SMA, fibronectin, and collagen type I. MAPK inhibitors (SB203580, SP600125) and NF-kB inhibitor (BAY11-7082) significantly decreased the protein expression associated with tissue remodeling (Fig 7A and 7B). These results suggest that apigenin alleviates TGF-β1-induced myofibroblast differentiation in nasal fibroblasts.

## Discussion

**Fig 8:**
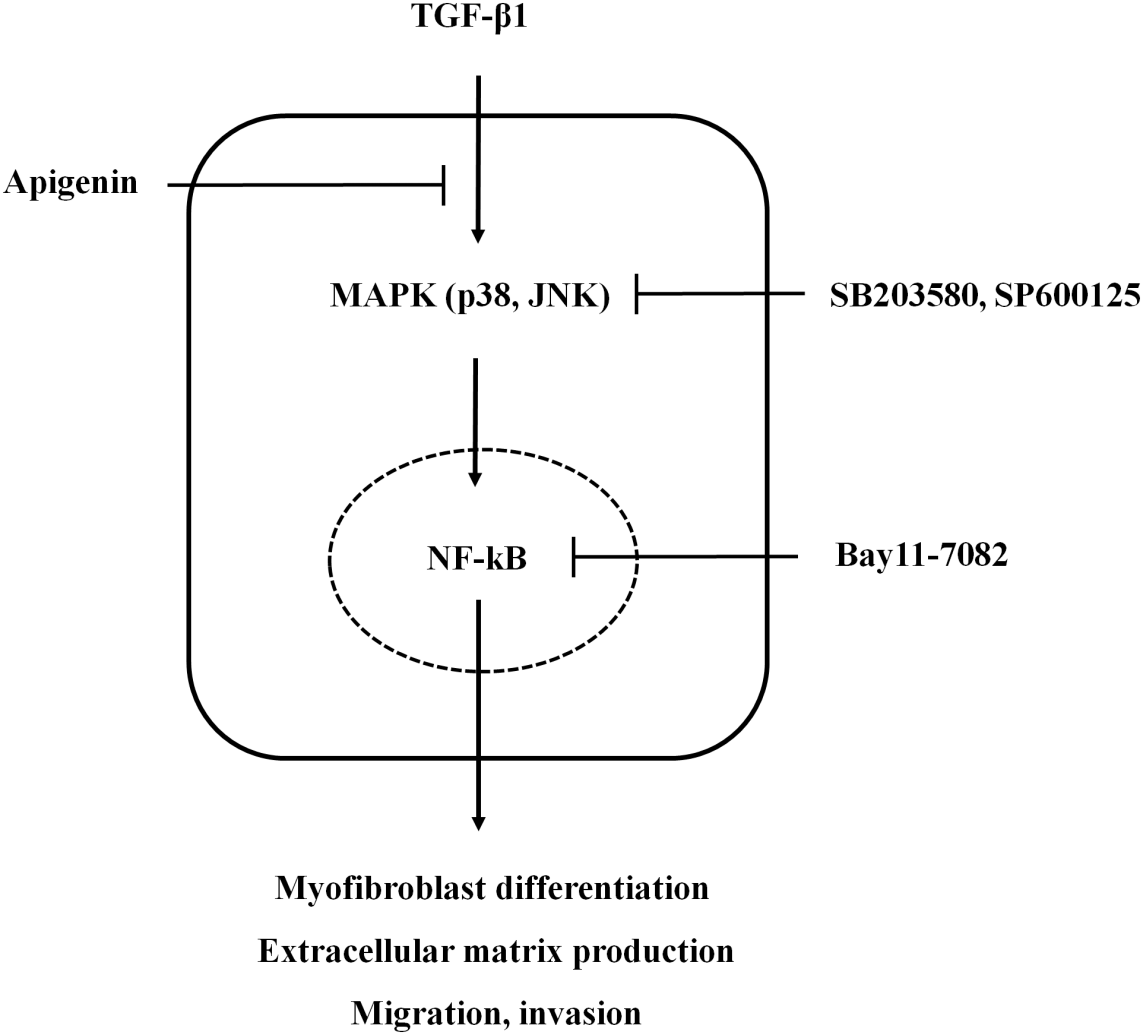
Schematic diagram of the effect of apigenin on TGF-β1-induced nasal fibroblast activation and extracellular matrix remodeling in nasal fibroblasts.

In this study, we evaluated the effect of apigenin on TGF-β1-induced fibroblast activation and ECM remodeling and its underlying mechanisms in nasal fibroblasts and inferior turbinate tissues. We found that treatment with apigenin suppressed TGF-β1-induced myofibroblasts and ECM by blocking the phosphorylation of MAPK and NF-κB pathways. Functionally, apigenin suppressed collagen contraction and cell migration in TGF-β1-induced nasal fibroblasts and *ex vivo* organ cultures of inferior turbinate (Fig 8).

CRS has two main forms; CRS without nasal polyp (CRSsNP) and CRS with nasal polyp (CRSwNP) [1, 18]. CRSsNP shows neutrophil infiltration and is characterized by glandular hyperplasia, myofibroblast differentiation, collagen deposition, and fibrosis. On the other hand, CRSwNP shows infiltration of eosinophils and edema and is known to be more severe than CRSsNP and recurrence rate of nasal polyps is high even after surgical removal [19]. However, the pathogenesis of CRS is still unclear.

Tissue remodeling is a process of remodeling or reconstructing an existing tissue and is generally known as a repair process that heals the wound through secretion and generation of an ECM when wounded [20]. However, excessive tissue remodeling can lead to pathogenesis of disease by inducing tissue fibrosis [21]. In the nose, activation of nasal fibroblasts induces ECM deposition and remodeling. It can be an important cause of CRSsNP [22]. The epithelial-to-mesenchymal transition in the stalk part or early stage of a nasal polyp in CRSwNP is involved in the overexpression of *a*-SMA, fibronectin, and collagen type I as well as the formation of nasal polyps and the cause of CRSwNP [6]. Therefore, tissue remodeling in CRS may be a major pathogenesis and therapeutic target.

TGF-β1, a representative profibrotic cytokine, has been reported to be a prime stimulator of fibroblast activation and to induce activation and differentiation of fibroblasts into myofibroblasts expressing α-SMA. TGF-β1 promotes massive amounts of ECM deposition, which leads to airway tissue remodeling [23, 24].

Apigenin is a bioflavonoid compound, and it affects cellular processes such as cell proliferation, migration, tumor growth, and fibrosis [12, 16, 25]. Ricupero *et al*. reported that apigenin inhibited the proliferation of myofibroblasts and expression of collagen type I and α-SMA and decreased TGF-β-induced α-SMA expression by inhibiting the Akt pathway in lung fibroblasts [26]. Jun *et al*. demonstrated that physiological concentrations of apigenin inhibited endothelin-1-induced contraction of collagen gels in an *in vitro* model of ECM remodeling [27]. In a bleomycin-induced systemic sclerosis mouse model, apigenin suppressed bleomycin-induced lung fibrosis [12].

We showed that apigenin has anti-tissue remodeling effects on TGF-β1 stimulated nasal fibroblast and inferior turbinate tissue. TGF-β1 stimulated nasal fibroblasts induced differentiation into myofibroblasts. Differentiated myofibroblasts increased the expression of ECM components such as fibronectin and collagen type I, leading to tissue remodeling. Apigenin partially reduced TGF-β1-induced total collagen expression at concentrations below 5 μM, but did not significantly decrease myofibroblast differentiation and inhibition of ECM components. However, apigenin at a concentration of 5 μM was found to have anti-tissue remodeling effects by significantly reducing a-SMA, fibronectin, and collagen type I. Apigenin inhibited the phosphorylation of MAPK (p38, JNK) induced by TGF-β1 and inhibited the production of ECM by blocking the transcription factor NF-κB pathway. We confirmed that the MAPK pathway regulates NF-κB by acting upstream of NF-κB through the specific antibodies of MAPK (SB203580, SP600125) and confirmed that myofibroblast differentiation and ECM synthesis are activated through this series of processes.

Activated fibroblast, or a myofibroblast, has mobility and collagen contractile activities. This functional role of the myofibroblast induces tissue remodeling [28]. Consequently, it is associated with pathogenesis of CRS. In the present study, we showed that treatment with apigenin suppressed enhancing cellular functions such as gel contraction and migration of nasal fibroblasts by TGF-β1, suggesting that apigenin has therapeutic potential for treatment of CRS.

Considering the above findings, we suggest that apigenin inhibits TGF-β1-induced tissue remodeling in nasal mucosa and inferior turbinate tissues via MAPK / NF-kB pathways and could contribute to the treatment and prevention of CRS.
